# Identification of Two Novel Wheat Drought Tolerance-Related Proteins by Comparative Proteomic Analysis Combined with Virus-Induced Gene Silencing

**DOI:** 10.3390/ijms19124020

**Published:** 2018-12-12

**Authors:** Xinbo Wang, Yanhua Xu, Jingjing Li, Yongzhe Ren, Zhiqiang Wang, Zeyu Xin, Tongbao Lin

**Affiliations:** 1 College of Agronomy, Henan Agricultural University, Zhengzhou 450002, China; boxinw@163.com (X.W.); yanhuaxu66@163.com (Y.X.); 13592589578@163.com (J.L.); wzq78@163.com (Z.W.); mrxxtz@163.com (Z.X.); 2 State Key Laboratory of Wheat and Maize Crop Science, Henan Agricultural University, Zhengzhou 450002, China; 3 Collaborative Innovation Center of Henan Grain Crops, Henan Agricultural University, Zhengzhou 450002, China; 4 Shangqiu Normal University, Shangqiu 476000, China

**Keywords:** drought stress, *Triticum aestivum* L., comparative proteomic analysis, iTRAQ, VIGS

## Abstract

Drought is a major adversity that limits crop yields. Further exploration of wheat drought tolerance-related genes is critical for the genetic improvement of drought tolerance in this crop. Here, comparative proteomic analysis of two wheat varieties, XN979 and LA379, with contrasting drought tolerance was conducted to screen for drought tolerance-related proteins/genes. Virus-induced gene silencing (VIGS) technology was used to verify the functions of candidate proteins. A total of 335 differentially abundant proteins (DAPs) were exclusively identified in the drought-tolerant variety XN979. Most DAPs were mainly involved in photosynthesis, carbon fixation, glyoxylate and dicarboxylate metabolism, and several other pathways. Two DAPs (W5DYH0 and W5ERN8), dubbed *TaDrSR1* and *TaDrSR2*, respectively, were selected for further functional analysis using VIGS. The relative electrolyte leakage rate and malonaldehyde content increased significantly, while the relative water content and proline content significantly decreased in the *TaDrSR1-* and *TaDrSR2*-knock-down plants compared to that in non-knocked-down plants under drought stress conditions. *TaDrSR1-* and *TaDrSR2*-knock-down plants exhibited more severe drooping and wilting phenotypes than non-knocked-down plants under drought stress conditions, suggesting that the former were more sensitive to drought stress. These results indicate that *TaDrSR1* and *TaDrSR2* potentially play vital roles in conferring drought tolerance in common wheat.

## 1. Introduction

As a result of the continuous growth of the global population and the adverse effects of environmental change on food production, food security has become a major problem worldwide [1]. Wheat (*Triticum aestivum* L.) is one of the most important food crops globally. At present, wheat production in many parts of the world is still dependent on excessive use of water, which is ecologically and economically unsustainable [2,3,4]. The shortage of water resources in many regions of the world greatly hinders crop productivity and sustainability. It is estimated that wheat production is likely to decline by 23% to 27% in 2050 due to global drought conditions [4,5]. Further exploration of the mechanisms underlying drought tolerance in wheat is therefore of vital importance for both the genetic improvement of drought tolerance in this crop and the reduction of the impact of drought stress on wheat production.

The physiological changes of wheat plants under drought stress, and the molecular mechanisms underpinning the response to drought stress have been well studied [6,7,8,9]. Relative water content (RWC), relative conductivity, malonaldehyde (MDA), and proline content are important physiological indicators that reflect plant drought tolerance. When subjected to water stress for extended durations, drought-tolerant varieties have been found to maintain a relatively higher RWC than drought-sensitive varieties [10,11]. Under severe drought stress conditions, membrane proteins are damaged, the membrane is easily broken, and even the cytoplasm is extravasated, resulting in an increase in relative conductivity [12,13]. The content of the membrane lipid peroxide MDA is an important marker of structural damage to the membrane [14]. Moreover, the accumulation of free proline under drought stress is positively correlated with drought tolerance in plants. Drought-tolerant varieties accumulate larger amounts of proline than drought-sensitive varieties [15]. A recent study in pepper reported a drought-inducible bZIP family gene, *CaDILZ1*, which controls plant drought tolerance by altering ABA content, stomatal closure, and the expression of ABA responses and drought response marker genes, while a RING-type E3 ligase, CaDSR1, can interact with CaDILZ1 and negatively regulate ABA signaling via E3 ligase activity, through influencing CaDILZ1 stability [16]. Studies have also shown that the AP2/ERF, MYB/MYC, and NAC transcription factors are associated with the drought stress response in wheat [17,18,19,20,21]. The expression of the AP2/ERF transcription factor-encoding gene *TaERF1* has been shown to be induced by drought stress. *TaERF1*-overexpressing transgenic *Arabidopsis*, wheat, and tobacco plants exhibit up-regulated expression of multiple stress response-related genes, which significantly enhances the tolerance of these transgenic plants to osmotic stress [22]. Overexpression of *TaMYB30* in *Arabidopsis* conferred significant resistance to drought during the germination and seedling stage [17,23]. The expression level of NAC transcription factor-encoding gene *TaNAC69* has also been demonstrated to be significantly up-regulated in response to drought stress. The overexpression of *TaNAC69* in wheat improves the drought tolerance of transgenic plants [24,25]. Peng et al. identified 93 (root) and 65 (leaf) drought/salinity stress-responsive proteins in a drought-tolerant somatic hybrid wheat cv. SR3 and its parent JN177. Most drought-responsive differentially abundant proteins (DAPs) also responded to salinity. The enhanced drought tolerance of SR3 appears to depend on the superior capacity for osmotic and ionic homeostasis, more efficient removal of toxic by-products, and better potential for growth recovery [26]. However, although extensive work has been conducted and significant progress in the elucidation of the physiological and molecular mechanisms underlying crop drought stress tolerance has been made [27,28], the mechanisms have not been fully explored, especially in hexaploid wheat.

In recent years, transcriptomics, proteomics, and other high-throughput research approaches have also been applied in the study of wheat tolerance to varying biotic and abiotic stresses [29,30]. In most cases, proteins are the ultimate functional molecules; therefore, proteomics has become a powerful and promising tool for the study of plant stress response [31]. However, although many drought-responsive proteins have been characterized [32,33,34,35,36,37], most of them have not been functionally verified. One important reason is that functional verification by transgenic research in some polyploid crops, especially in hexaploid wheat, is time-consuming and not suitable for high-throughput studies. Virus-induced gene silencing (VIGS) is an alternative strategy for gene functional analysis by simultaneously knocking down the expression of multiple gene copies, which can overcome the inherent problems of polyploidy and limited transformation potential that hamper functional validation studies in hexaploid wheat [38,39]. Currently, it has been used for the analysis of gene function in resistance against wheat pathogen and wheat aphid, as well as cold and drought stress [39,40,41,42,43]. In this study, the proteome profile of a drought stress-tolerant variety XN979 and a drought stress-sensitive variety LA379 were obtained under control and drought stress conditions, using the iTRAQ-based proteome approach. A total of 335 DAPs specifically identified in drought tolerance variety XN979 were obtained through a comparative proteomic analysis. Two DAPs (W5DYH0 and W5ERN8) were selected and dubbed *TaDrSR1* (drought stress response 1) and *TaDrSR2* (drought stress response 2), respectively, for further functional analysis using VIGS.

## 2. Results

### 2.1. Comparison of Drought Tolerance Between XN979 and LA379

**Figure 1:**
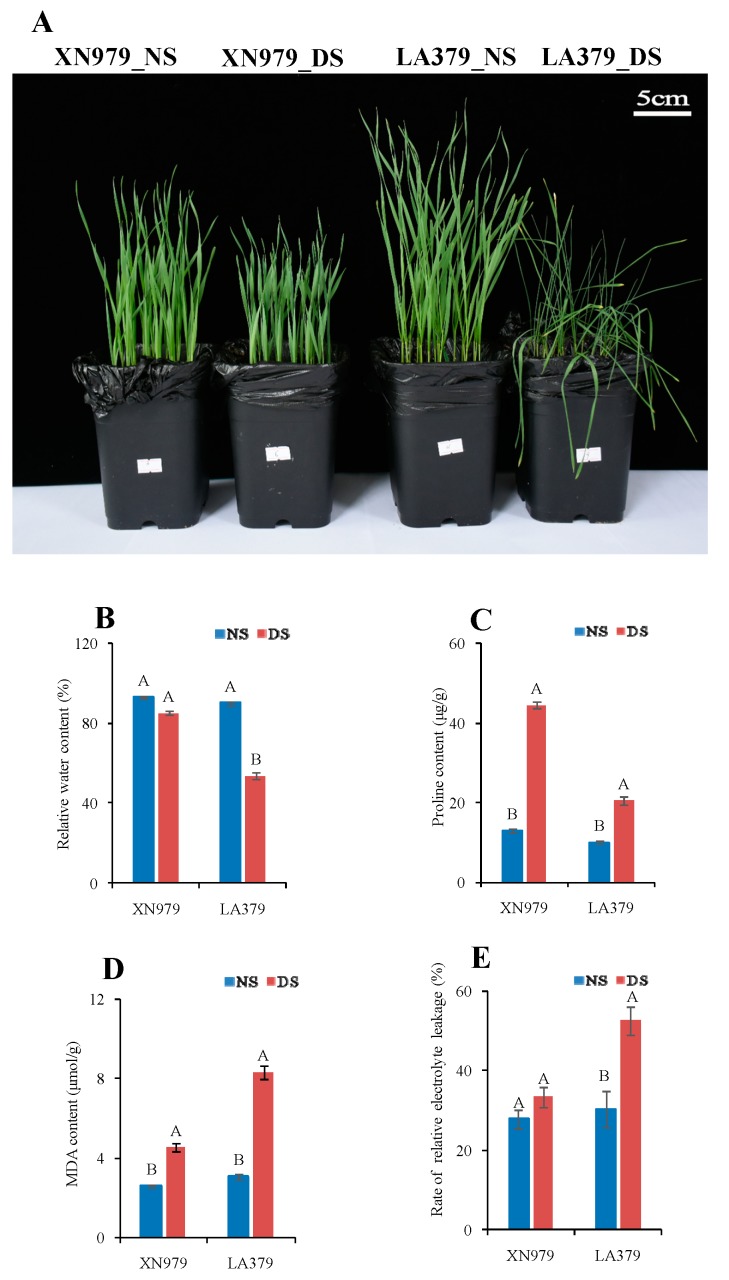
Phenotypic changes (**A**) and physiological responses (**B**–**E**) of XN979 and LA379 under drought stress (DS) and control (NS) conditions. (**A**) Phenotypic changes of XN979 and LA379 under drought stress (DS) and control (NS) conditions. (**B**) Relative water content (RWC); (**C**) Proline content; (**D**) malonaldehyde (MDA) content; (**E**) Rate of relative electrolyte leakage; NS, no stress; DS, drought stress. Three biological replicates were analyzed. Different letters indicate significant differences at *P* ≤ 0.01 levels.

As shown in Figure 1, 16 days after sowing, there were no significant phenotypic changes in drought-tolerant wheat varieties XN979 under drought stress (DS) conditions compared to no stress (NS) conditions. However, leaf drooping appeared in drought-sensitive wheat variety LA379 under drought stress conditions but did not under NS (Figure 1A). The RWC of LA379 dropped significantly by 40.39% but only dropped by 8.42% in XN979 (Figure 1B). As proline is an important osmotic regulator, we measured the content of proline in the leaves of both varieties under NS and DS conditions. Results showed that the content of proline significantly increased in both varieties: By 241.59% in XN979 under DS conditions relative to that under NS and 103.81% in LA379 (Figure 1C). MDA content and electrolyte leakage are important indices of membrane injury. The content of MDA in LA379 significantly increased by 169.64% under DS conditions relative to that under NS but only by 74.02% in XN979 (Figure 1D). Similarly, the electrolyte leakage was 73.05% higher than under NS in LA379 but only 20.05% higher than under NS in XN979 (Figure 1E).

### 2.2. Protein Identification and DAPs Analysis

**Figure 2:**
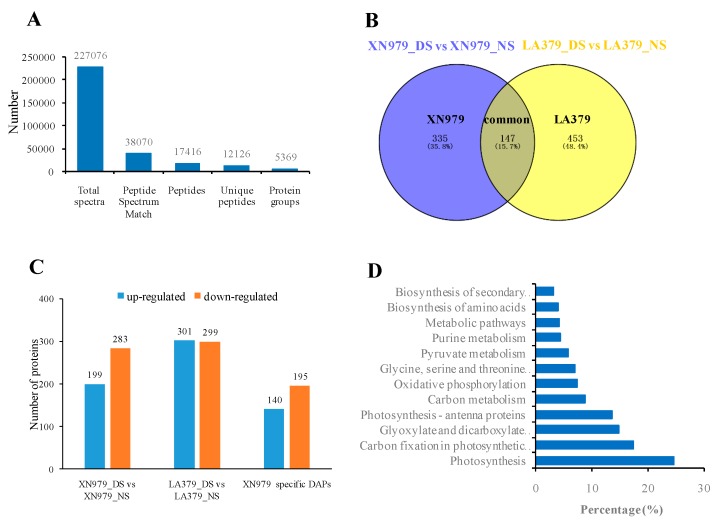
Statistical analysis of the proteome results and differentially abundant proteins (DAPs) under drought stress (DS) and control (NS) conditions. (**A**) Statistics for total spectra, matched spectra, matched peptides, unique peptides, and identified proteins. (**B**) Venn diagram analysis of DAPs in the one-to-one comparisons between NS and DS. (**C**) Number of up- and down-regulated DAPs in the XN979_DS-XN979_NS comparison, LA379_DS-LA379_NS, and drought-tolerant variety XN979 specific DAPs. (**D**) Enriched pathways of the DAPs specifically identified in XN979. The values on the abscissa indicate the percentage of the input number of DAPs among the number of the background proteins in the pathway; NS, no stress; DS, drought stress.

An iTRAQ-based comparative proteomic analysis was performed to identify wheat drought stress tolerance-related proteins. A total of 227076 spectra were generated and 38070 were matched to known spectra. These identified spectra were assigned to 17416 peptides, with 12126 unique peptides. Ultimately, 5369 proteins were identified (Figure 2A, Table S1). In the XN979_NS-XN979_DS comparison, a total of 482 proteins showed more than a 1.2-fold change (*P* < 0.05) in their respective abundances and were classified as DAPs (Table S2). Among the DAPs, 199 proteins were up-regulated and 283 proteins were down-regulated in XN979_DS compared to those in XN979_NS (Figure 2B,C). A comparison of LA379_NS and LA379_DS enabled the identification of 600 DAPs; these comprised 301 up-regulated and 299 down-regulated proteins (Figure 2B,C; Table S3). Among the DAPs identified above, 335 DAPs were exclusively identified in drought-tolerant variety XN979, but not in drought-sensitive variety XN979 (Figure 2B,C; Table S4). These 335 DAPs may be involved in the conferring drought stress tolerance in wheat.

### 2.3. Pathway Analysis of the DAPs Involved in Drought Stress Tolerance

To explore the metabolic pathways that the present DAPs were involved in, the 335 proteins were further investigated using the web-based tool KOBAS 3.0. In total, 12 pathways were significantly enriched in the 335 DAPs (Table S5, Figure 2D). The top four significantly enriched pathways were photosynthesis (24.7%), carbon fixation (17.4%), glyoxylate and dicarboxylate metabolism (14.9%), and photosynthesis-antenna proteins (13.6%). Other pathways, such as carbon metabolism, oxidative phosphorylation, and oxidative phosphorylation, may also be involved in drought stress tolerance (Table S5, Figure 2D).

### 2.4. Real-Time PCR Verification

**Figure 3:**
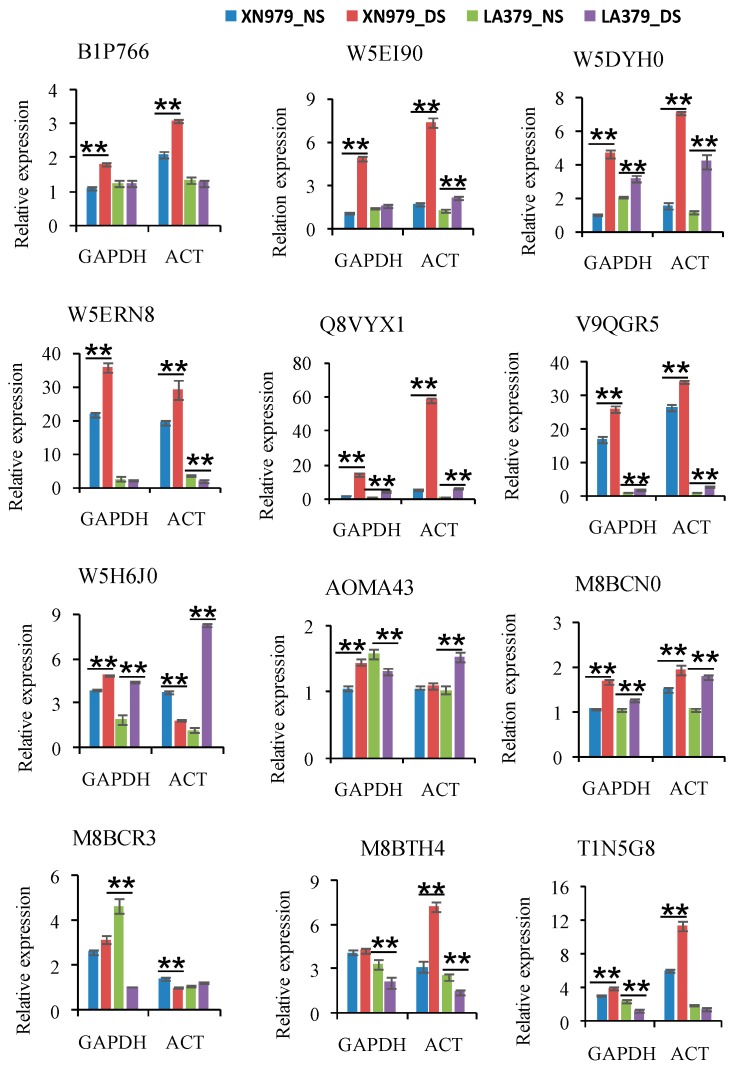
Relative mRNA expression analysis of twelve differentially abundant proteins (DAPs) using real-time PCR under drought stress (DS) and no stress (NS) conditions. Each bar shows the mean ± standard errors (SE) of three biological replicates. Two independent trials were conducted using *TaGAPDH* (*GAPDH*) and *TaActin* (*ACT*) as reference genes, respectively. The relative expression levels of each gene were calculated using the formula 2^−∆∆*Ct*^ (* *P* ≤ 0.05; ** *P* ≤ 0.01, Duncan’s multiple range test).

To verify the iTRAQ data and investigate the correlation of the abundance of proteins with their corresponding mRNA level, we randomly selected 4 XN979-specific DAPs (B1P766, W5DYH0, W5ERN8, and W5EI90), 5 LA379-specific DAPs (A0MA43, M8BCN0, M8BCR3, M8BTH4, and T1N5G8), and 3 common responsive proteins in both varieties (W5H6J0, V9QGR5, and Q8VYX1) for analysis of their RNA levels. *TaActin* and *TaGAPDH* were used as reference genes to normalize the expression level of target genes, respectively. Results showed that the expression levels of most DAPs were consistent with their protein expression levels (Figure 3). In most cases, the results of verification of expression using two different reference genes (*TaGAPDH* and *TaActin*) were similar. Only a few DAPs exhibited poor correlation between mRNA and protein expression levels (Figure 3).

### 2.5. VIGS of TaDrSR1 and TaDrSR2

**Figure 4:**
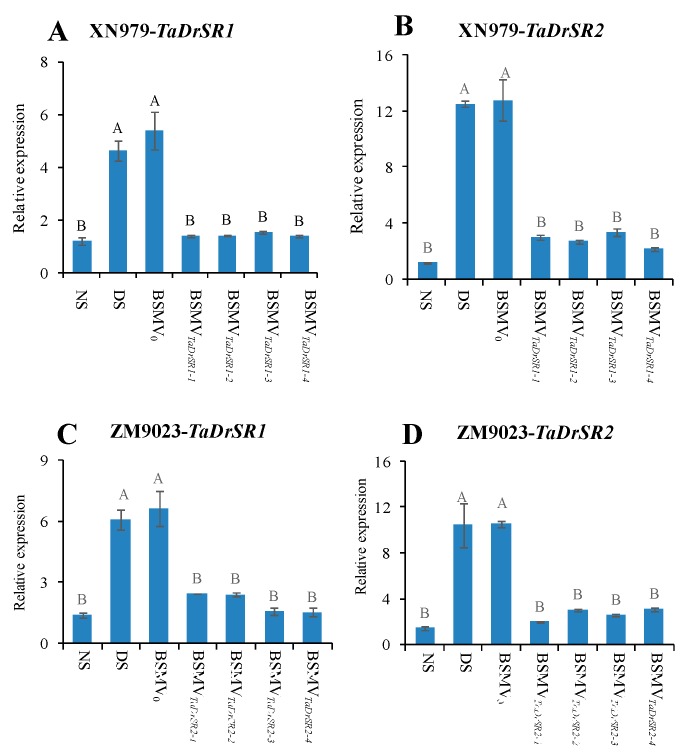
Detection of the expression levels of *TaDrSR1* and *TaDrSR2* in the corresponding knock-down plants. (**A**) The relative expression levels of the *TaDrSR1* gene in the *TaDrSR1*-knock-down plants of variety XN979. (**B**) The relative expression levels of the *TaDrSR2* gene in the *TaDrSR2*-knock-down plants of variety XN979. (**C**) The relative expression levels of the *TaDrSR1* gene in the *TaDrSR1*-knock-down plants of variety ZM9023. (**D**) The relative expression levels of the *TaDrSR2* gene in the *TaDrSR2*-knoc- down plants of variety ZM9023; NS, no stress; DS, drought stress. BSMV_0_, negative control of the virus-induced gene silencing (VIGS) system; BSMV*_TaD__rSR1_*, *TaDrSR1*-knock-down plants; BSMV*_TaD__rSR2_*, *TaDrSR2*-knock-down plants. Four independent *TaDrSR1-* and *TaDrSR2*-knock-down plants were analyzed, respectively. Different letters indicate significant differences at *P* ≤ 0.01 levels.

As mentioned above, the DAPs that were exclusively identified in drought tolerance variety XN979 are most likely involved in conferring drought stress tolerance. Therefore, we selected two functional uncharacterized proteins (W5DYH0 and W5ERN8) from the four qRT-PCR verified, XN979-specific DAPs (B1P766, W5DYH0, W5ERN8, and W5EI90) for further functional analysis using VIGS technology. Here, the drought stress-tolerant variety XN979 and another Chinese wheat variety ZM9023 were used for viral infection in the VIGS experiment independently. Firstly, we investigated the expression levels of *TaDrSR1* and *TaDrSR2* in four independent BSMV*_TaD__rSR1_*- and BSMV*_TaD__rSR2_*-infected plants; results showed that the transcription levels of the *TaDrSR1* and *TaDrSR2* genes were significantly knocked down compared to those of the negative control (BSMV_0_) and DS plants (Figure 4).

**Figure 5:**
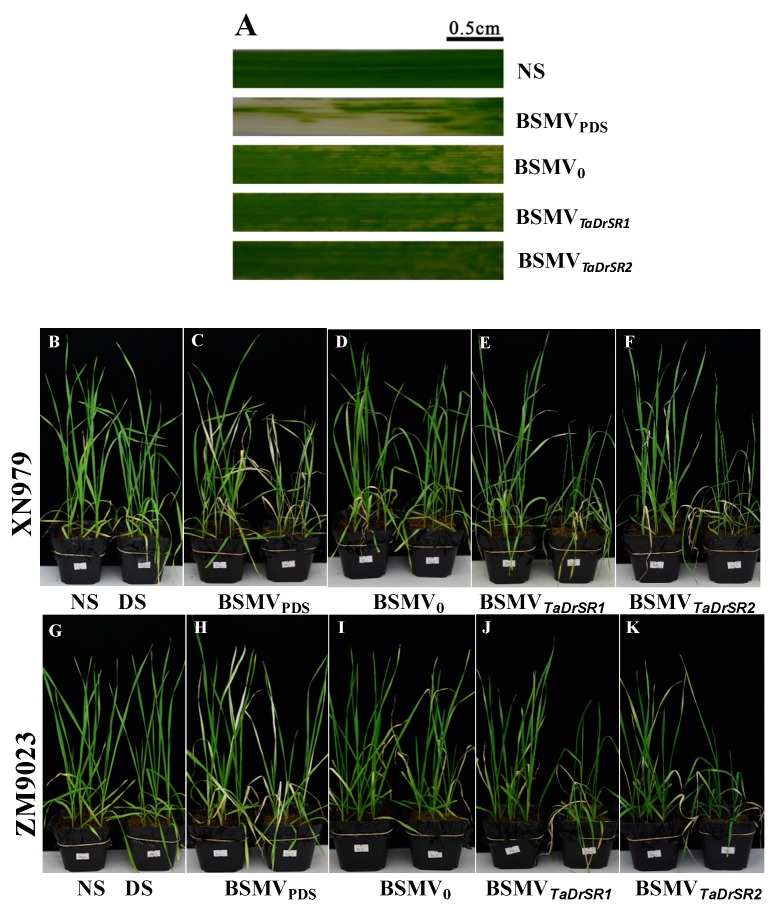
The phenotypes of *TaDrSR1-* and *TaDrSR2*-knock-down plants. (**A**) Leaf; (**B**–**K**) Whole plants; XN979 was selected as the receptor for viral infection in the VIGS experiment; (**G**–**K**) ZM9023 was selected as the receptor for viral infection in the VIGS experiment. The pot on the left side of each picture represents the no stress (NS) treatment and the pot on the right side represents the drought stress treatment (DS). BSMV_0_ represents the negative control of VIGS system; BSMV_PDS_ represents the positive control monitoring time course of VIGS; BSMV*_TaD__rSR1_* and BSMV*_TaD__rSR2_* represent *TaDrSR1-* and *TaDrSR2*-knock-down plants, respectively.

**Figure 6:**
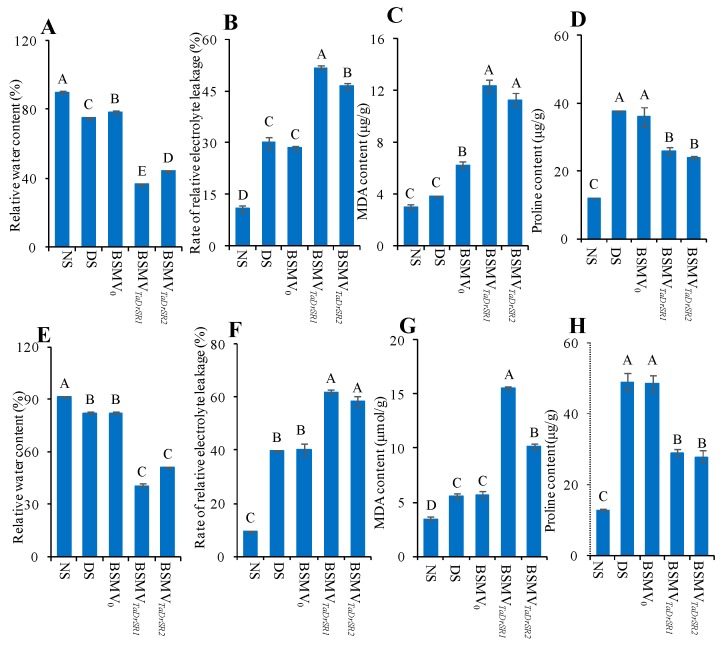
The changes in the physiological indices of the *TaDrSR1-* and *TaDrSR2*-knock-down plants. (**A**–**D**) XN979 was selected as the receptor for viral infection in the VIGS experiment; (**A**) relative water content; (**B**) rate of relative electrolyte leakage; (**C**) MDA content; (**D**) proline content. (**E**–**H**) ZM9023 was selected as receptor for viral infection in the VIGS experiment; (**E**) relative water content; (**F**) rate of relative electrolyte leakage; (**G**) MDA content; (**H**) proline content; NS, non-stressed plants; DS, drought-stressed plants; BSMV_0_, negative control of the VIGS system; BSMV*_TaD__rSR1_* and BSMV*_TaD__rSR2_*, *TaDrSR1-* and *TaDrSR2*-knock-down plants, respectively. Each bar shows the mean ± standard errors (SE) for three biological replicates. Different letters indicate significant differences at *P* ≤ 0.01 levels.

In XN979, ten days after infection, slight chlorosis was observed in all the BSMV construct-infected plants; this was attributed to the plant’s response to viral infection. The BSMV_PDS_-infected plants (positive control) exhibited bleached leaves (Figure 5A), indicating that the infection was successful [44]. Twenty days after infection, a substantial level of leaf drooping and wilting was observed in both BSMV*_TaD__rSR1_*- and BSMV*_TaD__rSR2_*-infected plants. However, no significant sagging and withering of the leaves was observed in plants infected with BSMV_0_ (negative control) and DS plants (Figure 5B–F). The leaf RWC in both BSMV*_TaD__rSR1_* and BSMV*_TaD__rSR2_*-infected plants reduced by 59.35% and 50.93% compared to that under NS but only reduced by 16.43% and 12.68% in the DS and BSMV_0_-infected plants (Figure 6A). The relative electrolyte leakage rate in both BSMV*_TaD__rSR1_*- and BSMV*_TaD__rSR2_*-infected plants increased by 371.75% and 322.62% compared to that under NS, respectively, which was much higher than that in the DS and BSMV_0_-infected plants (Figure 6B). MDA content in both BSMV*_TaD__rSR1_*- and BSMV*_TaD__rSR2_*-infected plants increased by 314.08% and 277.34% compared to that under NS respectively, which was significantly higher than that in the DS and BSMV_0_-infected plants (Figure 6C). Proline content in both BSMV*_TaD__rSR1_*- and BSMV*_TaD__rSR2_*-infected plants increased by 114.89% and 98.43% compared to that under NS, respectively, which was significantly lower than that in the DS and BSMV_0_-infected plants (Figure 6D). Similar results were obtained for ZM9023 (Figure 5G–K and Figure 6E–H).

## 3. Discussion

Drought stress occurs frequently worldwide and is a major limiting factor for plant growth and productivity. It has been reported that wheat can adapt in response to drought stress and exhibit significant differences in drought tolerance among different genotypes [45]. The elucidation of the molecular mechanisms and investigation of drought tolerance-related genes/proteins is crucial for the genetic improvement of plant drought tolerance.

Comparative proteomic analysis is a powerful tool for the study of plant stress response [31,46]. The changes in protein expression profiles under drought conditions have been investigated in several plants, including wheat, maize, rice, peanut, and soybean, and many drought-responsive proteins have been characterized [32,33,34,35,36,37]. However, despite this progress, most of these proteins have not been functionally verified. The molecular mechanism underlying plant drought tolerance is still far from fully elucidated, especially in hexaploid wheat. Here, a comparative proteomic analysis of two wheat varieties with contrasting drought tolerance enabled the identification of 335 DAPs specific to the drought-tolerant variety XN979, and 147 common responsive DAPs in both varieties (Table S4, Figure 2B). The XN979-specific drought-responsive proteins may participate in pathways mediating drought stress tolerance in wheat. Pathway enrichment analysis indicated that most of these proteins were involved in photosynthesis, carbon fixation, glyoxylate and dicarboxylate metabolism, and some other pathways (Figure 2D). In our study, 19 DAPs involved in the photosynthesis pathway were significantly enriched in drought-tolerant variety XN979. Previous studies have shown that the photosynthesis pathway is indeed affected by drought stress. The photosynthetic rate and the assimilation product are reduced, thereby further reducing the material basis of leaf growth [26,47,48,49]. The photosynthetic rate and the chlorophyll a content in a drought-tolerant variety SR3 were higher than in the leaves of its parent JN177. This higher capacity to maintain photosynthesis under stress may be achieved through a combination of a more robust cellular homeostasis and a more effective means of removing ROS and other toxic by-products [26]. Therefore, the 19 DAPs involved in the photosynthesis pathway may play important roles in drought tolerance in wheat. Other pathways, such as carbon fixation and photosynthesis-antenna proteins, are also associated with plant photosynthesis [50]. The other enriched pathways, such as carbon metabolism, pyruvate metabolism, amino acids metabolism, and oxidative phosphorylation, are mainly involved in the metabolism and allocation of carbohydrates. These results indicate that the regulation of the photosystem and carbohydrates metabolism play important roles in wheat drought tolerance.

Two DAPs (W5DYH0 and W5ERN8) were selected and dubbed *TaDrSR1* and *TaDrSR2*, respectively, for further functional analysis using VIGS technology. The ortholog of *TaDrSR1* in rice, r40c1, is up-accumulated in the roots of *DREB1A* transgenic plants, which may play an important role in the generation of drought-tolerant plants [51,52]. *TaDrSR2* encodes a functional unknown protein with 71 amino acid residues. Therefore, we know very little about the functions of both genes. We analyzed the chromosomal locations of *TaDrSR1* and *TaDrSR2* according to Yang et al. [53] and found that *TaDrSR1* and *TaDrSR2* were anchored on chromosome 4A and 2D, respectively. Interestingly, the chromosomal regions also have a number of QTLs for agronomic traits under drought stress conditions [54,55,56]. For example, a QTL for the root number under water-limited environments, *qRN.qgw-4A*, is linked with the *TaDrSR1* gene [54]. This chromosomal region also harbors QTLs for the drought stress susceptibility index (SSI) and drought stress tolerance (TOL) [56]. *TaDrSR2* is also linked with QTLs for plant height, spike length, spikelets per spike, and kernels per spike under drought stress conditions [55]. This evidence further confirms that the chromosomal regions in which *TaDrST1* and *TaDrST2* are located are important for wheat drought stress tolerance. As RWC, relative conductivity, MDA, and proline content are important physiological indicators reflecting plant drought tolerance [14,57,58,59], we measured these indices in the *TaDrSR1-* and *TaDrSR2*-knock-down plants and negative controls. Our results showed that the relative electrolyte leakage rate and MDA content significantly increased, while the RWC and proline content significantly decreased in the VIGS-mediated *TaDrSR1-* and *TaDrSR2*-knock-down plants compared to that in the controls (BSMV_0_ and DS) under drought stress conditions (Figure 6). The *TaDrSR1-* and *TaDrSR2*-knock-down plants exhibited severe drooping and wilting phenotypes relative to the negative control (BSMV_0_-infected plants) and non-infected plants under drought stress conditions, indicating that they were more sensitive to drought stress (Figure 5B–F). We performed the VIGS trial using another variety (ZM9023) as receptors for viral infection and obtained similar results (Figure 5G–K and Figure 6E–H). Previous studies have shown that the higher the MDA content of plants, the greater the level of membranous peroxidation and permeability. Further, the cytoplasm undergoes extravasation under drought stress, resulting in an increase in relative conductivity [14]. Under drought stress, proline reduces the osmotic potential, plays a role in osmotic regulation, and protects cell membrane structure; these changes represent an adaptive response of plants to adverse environments [60,61,62]. Our results, together with those of previous studies, strongly suggest that *TaDrSR1* and *TaDrSR2* potentially play vital roles in conferring drought tolerance in common wheat.

A comparative proteomic analysis approach can identify hundreds of wheat drought-tolerant protein candidates. For example, in this study, we identified 335 drought tolerance-related proteins, which were involved in 12 pathways (Figure 2D; Table S5). Some of them may play important roles, while others may only serve as an aid. The verification the function of these proteins is a big challenge in hexaploid wheat. VIGS has the advantages of simple operation and short test period [39,40]. It is suitable for preliminary functional verification on a large number of candidates. Therefore, comparative proteomic analysis combined with VIGS is a good choice for identifying novel genes for drought tolerance in wheat, although subsequent transgenic validation is necessary.

In summary, we identified 335 DAPs involved in wheat drought tolerance. Most of them were involved in the pathways of photosynthesis, carbon fixation, glyoxylate and dicarboxylate metabolism, carbon metabolism, and oxidative phosphorylation. Two DAPs *TaDrSR1* and *TaDrSR2* were selected for further functional analysis using VIGS. The *TaDrSR1*- and *TaDrSR2*-knock-down plants exhibited severe drooping and wilting phenotypes under drought stress conditions. Further physiological and molecular analyses indicated that these knock-down plants were more sensitive to drought stress, suggesting that *TaDrSR1* and *TaDrSR2* potentially play vital roles in conferring drought tolerance in common wheat. Our results also showed that comparative proteomic analysis combined with VIGS is an efficient way for identification of novel drought tolerance-related proteins in hexaploid wheat.

## 4. Materials and Methods

### 4.1. Plant Materials

XN979 and LA379, two wheat varieties with contrasting drought tolerance, were used as materials to conduct comparative proteomic analysis. XN979 exhibits far higher tolerance to drought stress than LA379. The drought stress-tolerant variety XN979 was used for viral infection in the VIGS experiment. A previous study reported that ZM9023 is susceptible to viral infection in VIGS trials [39]. To further confirm the results of VIGS in XN979, ZM9023 was also selected as a receptor for viral infection in this study.

### 4.2. Plant Growth Conditions and Sampling

Pot culture was used in this trial. Firstly, seeds were disinfected with 1% H_2_O_2_ for 10 min and then rinsed with distilled water for three times. Before sowing, substrate (Pindstrup Substrate Peat) and vermiculite were well mixed (volume ratio 1:2). Seeds were germinated for 16 h at 22 °C; then, 24 germinated seeds were sown in each pot with soil water content at 90% field capacity (FC). The pots were divided into two groups for two watering treatments, respectively: (i) A group of 20 pots (10 pots per cultivar) was grown under well-watered conditions (maintained at 80–90% FC, NS), and (ii) another 20 pots (10 pots per cultivar) were subjected to drought stress (DS, no watering after sowing). The temperature ranged from 22–23 °C in the daytime and 18–20 °C at night during the pot culture trial. Wheat plants were thinned to 20 plants per pot after emergence. Sixteen days after sowing (about 44% FC under DS conditions), fresh leaves of XN979 and LA379 under NS and DS conditions were sampled, respectively, and used for the measurement of physiological indices (proline, MDA content, and electrolyte leakage), with at least three biological replicates. The leaves of fifteen individual plants of XN979 and LA379 under NS and DS conditions were mixed respectively, fast-frozen in liquid nitrogen, and stored in a −80 °C freezer for protein extraction. This collection was repeated for the second biological replicate. The samples of two biological replicates of XN979 and LA379 under NS and DS conditions were named as XN979_NS-1, XN979_NS-2, LA379_NS-1, LA379_NS-2, XN979_DS-1, XN979_DS-2, LA379_DS-1, and LA379_DS-2, respectively. For RNA extraction and real-time PCR verification, three biological replicates were analyzed. Twenty days after sowing (about 40% FC under DS conditions), the leaves were collected for measurement of RWC.

### 4.3. The Measurement of Physiological Indices

The RWC, which was measured as described by Flexas et al. [63], was calculated based on the following formula: RWC (%) = [(FW − DW)/ (TW − DW)] × 100; FW represents fresh weight, TW refers to turgid weight, and DW represents dry weight. Electrolyte leakage was assayed according to Yan et al. [43]. Proline was extracted and determined according to the method of Bates et al. [64]. MDA content was measured following the methods of Hodgeset al. [65].

### 4.4. Protein Extraction, Digestion, and iTRAQ Labeling

Protein extraction was performed according to Thiellementet al. [66]. Protein digestion was performed using the FASP procedure [67]. Finally, a peptide mixture (100 μg) of each sample was labeled with iTRAQ reagents according to the manufacturer’s instructions (Applied Biosystems, USA). XN979_NS-1, XN979_NS-2, LA379_NS-1, LA379_NS-2, XN979_DS-1, XN979_DS-2, LA379_DS-1, and LA379_DS-2 were labeled with 113, 114, 115, 116, 117, 118, 119 and 121, respectively.

### 4.5. Strong Cationic Exchange (SCX) Fractionation and LC–ESI–MS/MS Analysis

iTRAQ labeled peptides were combined and dried under vacuum. Strong cationic exchange (SCX) chromatography was performed to fractionate the labeled peptides using the AKTA Purifier system (GE Healthcare) as previously described, with minor modifications [68,69]. In brief, the labeled peptide mixture was acidified using 10 mM KH_2_PO_4_ in 25% of ACN (buffer A, pH 3.0) and eluted at a flow rate of 1 mL/min with a gradient of 0–8% 500 mM KCl, 10 mM KH_2_PO_4_ in 25% of ACN (buffer B, pH 3.0) for 22 min, 8–52% buffer B for 22–47 min, 52%–100% buffer B for 47–50 min, 100% buffer B for 50–58 min, and buffer B was reset to 0% after 58 min. The elution was monitored by absorbance at 214 nm, and fractions were collected every 1 min. Samples were reconstituted with trifluoroacetic acid and stored at −80 ◦C for LC–MS/MS analysis.

Liquid chromatography–electrospray ionization tandem MS analysis was performed using a Q Exactive mass spectrometer coupled to an Easy nLC (Proxeon Biosystems) according to previously reported literature with minor modifications [68,69]. The peptide mixture was loaded onto a reverse phase trap column connected to the C18-reversed phase analytical column in 0.1% formic acid (buffer A) and separated with a linear gradient of 0.1% formic acid and 84% acetonitrile (buffer B) with a 300 nL/min flow rate. The linear gradient was 0–35% buffer B for 0–50 min, 35–100% buffer B for 5 min, and hold in 100% buffer B for another 5 min. The most abundant precursor ions were dynamically selected from the survey scan (300–1800 m/z) to acquire MS data. The instrument was run with the peptide recognition mode enabled [68,69].

### 4.6. Data Analysis

MS/MS spectra were searched using MASCOT engine (Matrix Science, version 2.2) embedded into Proteome Discoverer 1.4 and run against the UniProt_Poaceae database and the decoy database (http://www.uniprot.org). The Mascot search parameters were set according to previous studies [68,69]. Mascot search parameters were set as follows: Enzyme: Trypsin; max missed cleavage: 2; fixed modification: Carbamidomethyl (C), iTRAQ8plex(N-term), iTRAQ8plex(K); variable modification: Oxidation (M), iTRAQ8plex (Y); peptide mass tolerance: ± 20 ppm; fragment mass tolerance: 0.1 Da; peptide false discovery rate (FDR) ≤ 1%. All peptide ratios were normalized by the median protein ratio. The abundance of each protein was calculated as the median of unique peptides of the protein, and the fold change was defined based on the abundance of the protein under drought relative to their respective level under control. Protein species with an abundance ratio fold change of at least 1.2 and a *P*-value < 0.05 were defined as DAPs [70,71].

### 4.7. Pathway Enrichment Analysis

Pathway enrichment analysis was performed using a web-based tool KOBAS 3.0 (http://kobas.cbi.pku.edu.cn/anno_iden.php) [72]. The adjusted *P*-values with Benjamini–Hochberg correction under a threshold of 0.05 were considered to represent statistically significant differences.

### 4.8. Quantitative Real-Time PCR

Total RNA from the leaves was extracted using the total RNA kit (TaKaRa, Dalian, China). The Two-Step Prime-Script TM RT Reagent Kit (Perfect Real Time; TaKaRa) with gDNA Eraser was used for the reverse transcription reactions following the manufacturer’s instructions. Twelve DAPs were randomly selected for RNA-level examination. The specific primers were designed using Primer 5.0 (Premier Biosoft, Palo Alto, CA, USA). The cDNA samples were used as templates and mixed with primers and SYBR Premix ExTaq (TaKaRa) for real-time PCR analysis. Real-time PCR was conducted using a BioRad IQ5 Real-time PCR Detection System (Bio-Rad, Hercules, CA, USA). The temperature settings were 95 °C for 5 min followed by 40 cycles of 95 °C for 15 s, 60 °C for 15 s, and 72 °C for 15 s. *TaActin* and *TaGAPDH* were used as reference genes to normalize the expression level of target genes, respectively. Relative gene expression was computed using the 2^−^^ΔΔ*Ct*^ method [73]. All primers used in this study are listed in Table S6.

### 4.9. Vector Construction for VIGS

To further study the functions of DAPs, we constructed VIGS vectors of *TaDrSR1* and *TaDrSR2*. Firstly, 118 bp- and 107 bp-fragments of the coding regions of *TaDrSR1* and *TaDrSR2* were cloned, respectively. Vector construction was performed as previously described [74]. Linearized plasmids were used as templates to synthesize α, β, and γ RNAs of the BSMV genome with the RiboMAX TM Large Scale RNA Production System-T7 (Promega, Madison, WI, USA) [75]. In vitro transcripts of each RNA fragment were mixed in an equimolar ratio and added to triturated FES buffer [40]. Then, the mixture was diluted and treated with an equal volume of DEPC. Each of the silencing constructs consisted of BSMV α, β, and γ with the target gene insertion. The original BSMV_0_ was constructed from α, β, and γ derived from the original empty pSL038-1 vector and used as the negative control. BSMV_PDS_ (in which the wheat gene encoding phytoene desaturase, GenBank: FJ517553.1, was silenced) was used as a positive control of VIGS [41]. BSMV*_TaD__rSR1_* was constructed from α, β, and γ with the insertion of the target gene *TaDrSR1*. BSMV*_TaD__rSR2_* was constructed from α, β, and γ with the target gene *TaDrSR2*’s insertion.

### 4.10. Infection with VIGS Vectors

The drought stress-tolerant variety XN979 and virus-susceptible variety ZM9023 were selected as receptors for infection with VIGS vectors. Two-leaf-stage plants of XN979 and ZM9023 were used for infection, which was performed according to previously described procedures [40]. After the infection, the incubator temperature was set at 23 ± 2 °C, with darkness for 24 h, followed by a 16 h light/8 h dark photoperiod. Twenty days after the infection, the third and fourth leaves were collected and stored at −80 °C for measurement of physiological indices (proline and MDA content) and real-time PCR analysis. Twenty-four days after the infection, the leaves of the remaining plants were collected for the measurement of RWC and rate of relative electrolyte leakage.

### 4.11. Data Analysis

The means, standard errors (SE), and ranges of each measured morphological trait were analyzed using IBM SPSS statistics 21 software. For each morphological trait and gene expression level, *P* = 0.05 and *P* = 0.01 were used as thresholds to identify significant and extremely significant differences, respectively (Duncan’s multiple range test). Venny 2.1 (a web-based tool) was used to generate Venn diagrams (http://bioinfogp.cnb.csic.es/tools/venny/index.html).

## Supplementary Materials

Supplementary materials can be found at http://www.mdpi.com/1422-0067/19/12/4020/s1. All data related to this study have been deposited into the publicly accessible database iProX (ww.iprox.org) with identifier IPX0001326000/PXD011115.

## Abbreviations

VIGS	Virus-induced gene silencing
DAPs	Differentially abundant proteins
MDA	Malonaldehyde
RWC	Relative water content
TaDrSR1	Drought stress response 1
TaDrSR2	Drought stress response 2
DS	Drought stress
NS	No stress
FC	Field capacity
SCX	Strong cationic exchange
BSMV	Barley stripe mosaic virus
FASP	Filter aided sample preparation
ACN	Acetonitrile
